# nAMD: optimization of patient care and patient-oriented information with the help of an internet-based survey

**DOI:** 10.1007/s00417-022-05678-7

**Published:** 2022-05-13

**Authors:** Anke Schmid, Felicitas Bucher, Erika Liczenczias, Sara Maslanka Figueroa, Bettina Müller, Hansjürgen Agostini

**Affiliations:** 1 Eye Center, Faculty of Medicine, University of Freiburg, Freiburg, Germany; 2 Novartis Pharma GmbH, Nuremberg, Germany

**Keywords:** Age-related macular degeneration, Survey, Internet-based, Quality of Life, SF-12v2, nAMD

## Abstract

**Purpose:**

This survey was conducted to identify factors that influence how patients with neovascular age-related macular degeneration (nAMD) deal with their disease and information that are considered useful from a patient’s point of view.

**Methods:**

A total of 5035 patients with nAMD living in Germany were interviewed via internet-based cross-sectional survey, where the following information was collected: personal data, disease awareness, and patients’ needs. In addition, a Quality of Life questionnaire (SF-12v2) could be completed.

**Results:**

Out of the 5035 participants, more males than females participated (55% vs 45%), and most participants were in the age groups 76 to 85 years (37%) and 66 to 75 years (35%). Seventy-three percent of patients rated their understanding of the disease as at least sufficient, and more than two-thirds of the patients (68%) were aware that their disease needs to be controlled on a regular basis and treated on an “as needed” basis. Regarding potential risk factors for AMD, most participants were aware of age (89%), but only 39% of hereditary load and 33% of smoking as evidence-based risk factors, indicating a need for further information. The doctor remains the major source of information (93%), with internet (29%), brochures (14%), opticians (13%), or patient support groups (4%) with only limited contribution. Distance to the treatment center was identified as one of the factors, which had the greatest influence on patients’ compliance. A “treat as needed” regime turned out to be the preferred control and treatment schedule in contrast to a “fixed appointment” every 4 weeks.

**Conclusion:**

This internet-based survey appears to be representative for nAMD patients. To increase patients’ compliance, proximity to the treatment center and a “treat as needed” regime turned out to be important factors as well as patients’ awareness of their disease. In this regard, the reported desire for more information indicates that patients’ knowledge still needs to be improved. Our results will help to further optimize patient care and patient-oriented information.

**Supplementary Information:**

The online version contains supplementary material available at 10.1007/s00417-022-05678-7.


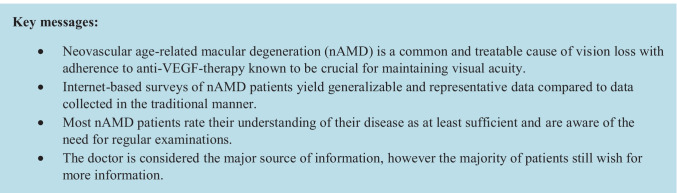


## Introduction

Age-related macular degeneration (AMD) is the main cause of blindness in industrial nations. It is estimated to cause about 9% of all cases of blindness worldwide and its prevalence has risen markedly. For instance, in Germany, the number of early AMD rose from 5.7 million in 2002 to around 7 million in 2017 [1, 2].

About 10–20% of AMD patients progress into an exudative form of AMD (neovascular AMD, nAMD) with the development of pathological blood vessels during the course of the disease [2]. More than 10 years ago, the establishment of anti-VEGF therapy has revolutionized the treatment of patients suffering from nAMD in a groundbreaking way. Since then, the blindness rate of this patient group has been reduced by about 50% [3–5]. At the same time, it has become clear that the therapeutic success and long-term stability of visual acuity strongly depend on continuous treatment of nAMD patients due to the chronic nature of the disease [4].

Although, there are some discrepancies between results of clinical trials and real-life evidence [5], there is an agreement in both that better disease control is associated with greater improvements in visual acuity [6]. While initial visual acuity improvement is maintained for at least 2 years in pivotal studies [7–9], in real-life settings, it often declines over time. However, real-life clinical outcomes also showed a significant correlation between the increase in visual acuity ≥ 3 lines and patient compliance after the first year of treatment [10]. This positive effect was observed up to 2 years in the observational study by Holz et al. [6], where there was a positive correlation between the number of examinations (ophthalmoscopies, OCT) and visual acuity. Compared to clinical trials, patients in real-life settings enter treatment with better initial visual function but receive fewer injections after the initial treatment phase [11]. It has been shown that a lower number of annual injections is directly related to poor visual outcome [11, 12]. Therefore, patient adherence to anti-VEGF therapy seems to play a key role to ensure continuous monitoring and adequate treatment. Delayed first treatment can have a significant negative impact on visual acuity. Such effects can even be observed after a delay of only 2 weeks [4]. Therefore, the time between diagnosis and start of the treatment is another important factor to control the disease [4].

Data from several studies show that 19–50% of nAMD patients turn out to be non-adherent [13–15]. The observational cohort study by Ehlken et al. [10] analyzed treatment adherence and real-life clinical outcomes within the first year of treatment of patients with nAMD, diabetic macular edema (DME), and macular edema secondary to retinal vein occlusion. The investigators reported that 32% of nAMD patients and 44% of DME patients requiring treatment turned out to be non-adherent [10].

Reasons for non-adherence may include poor access to an ophthalmologist due to reduced mobility, organizational problems with appointments, and comorbidities of the patient [16, 17]. Additionally, the fear of injections has also been described as a reason for insufficient therapy compliance [18, 19].

For the above-mentioned reasons, it is clear that there is medical need to increase patient adherence to intravitreal anti-VEGF therapy. It is of utmost importance to identify the critical factors hampering compliance (e.g., reasons for not keeping appointments, differences in care due to the insurance situation, optimal control and treatment regimen) in order to ensure patient adherence and long-lasting success of the therapy. Therefore, the internet-based data collection survey, OPTIMA, was initiated to gain a deeper understanding of the behavior of nAMD patients by questioning them about their understanding of the disease (awareness), their perception of the course of treatment, and their use of the internet to search for information about their disease. In addition, patients were able to complete the SF-12v2 Quality of Life (QoL) questionnaire to assess how satisfied they are with their life situation.

OPTIMA aimed at collecting data concerning the “journey” of patients with nAMD, giving important insight about patients’ subjective perception of the disease and its course of treatment. Results related to patients’ level of knowledge and information acquisition will contribute to the optimization of the most accepted sources of information for both patient education and care, as well as study-related patient support tools.

With the help of this survey, we tried to identify not only factors that influence how patients deal with their disease but also the kind of information that is considered useful from a patient’s point of view.

## Methodology

The study was conducted by means of an internet-based cross-sectional survey.

Participation in the OPTIMA internet-based data collection was voluntary and anonymous. Patients had to actively consent to participation through clicking a separate agreement button. All patient data collected were recorded in anonymized form using a secure server. Interested people and participants of the survey were informed about the privacy policy before starting the survey. The results of the survey were evaluated in an aggregated form.

It was planned to interview 5000 patients with nAMD living in Germany. When considering between 29 to 48 thousand first treatments for nAMD in 2015 in Germany [1, 20], we assumed that 10% of these should be a representative sample size for the OPTIMA survey.

The initial planned survey accessibility period for potential participants was 4 months starting in May 2020. Once the target number of participants was reached, the questioning period was closed, and the questionnaire removed. Approximatively 20–30 min were needed to answer the survey completely and each participant could respond to the questions only once. This was assured by setting a cookie that prevented re-participation. Beyond that, no further procedures could be implemented, as this was an open and anonymous survey.

The survey was implemented in a user-friendly screen reader on a barrier-free website with a larger font size (28 pixel) for better readability. The survey was advertised on Google Display, social networks (Facebook), and the search engine Google. Interested parties could access the ClinLife survey platform by clicking on the information/advertisement button. Study participants were recruited exclusively online and institutions did not provide information about the survey to their patients. Furthermore, the study was conducted on a neutral website without any logos or information about the sponsoring company.

Of central importance to the survey was the analysis of patients’ needs, barriers, and preferences concerning the anti-VEGF treatment of nAMD. This particularly included the identification of the main reasons for non-attendance of appointments. It was also important to know how, from the patient’s perspective, an optimal monitoring and treatment regimen could be designed to improve patient compliance. In addition, it was tried to clarify if there were differences in care due to patient insurance (private vs statutory).

The following information was collected in the survey (for the complete questionnaire consisting of 31 questions (without SF12v2), please refer to the supplementary information):

- Personal data: age in years, gender, country of residence, confirmed nAMD, health insurance.
- Disease awareness: patient’s knowledge about progression and risk factors, including sources of information used.
- Patient’s history: visual symptoms, duration of disease, type of diagnostics, diagnosis, treatments, care in the ophthalmology practice, care expenditure for ophthalmologist visits.
- Patient’s needs: best monitoring and treatment regimen, further need for information.

For all questions, answer options were given from which the patient could choose, or time frames could be entered.

The validated standardized Quality of Life (QoL) questionnaire SF-12v2 was used to find out how satisfied patients with nAMD are with their health-related quality of life. The SF-12v2 questionnaire is a shortened version of the SF-36 questionnaire and is considered a valid instrument in ophthalmological research for assessing the general state of health. It consists of two questions each from the domains Emotional Role Functioning, Psychological Well-being, Physical Functioning, and Health-related Quality of Life, and one question each related to General Health Perception, Vitality, Physical Pain, and Social Functioning [21].

### Statistics

The parameters collected in this survey were primarily descriptive. For quantitative parameters, the mean value and standard deviation and minimum and maximum as well as the quartiles including the median were given for the total collective as well as for subgroups. Qualitative parameters were described by means of absolute and percentage frequencies, and subgroups were compared using contingency tables. Statistical tests were performed bilaterally, based on a 5% significance level. Alpha adjustment for multiple testing was not considered necessary due to the descriptive nature of this study.

## Results

The initial planned survey runtime was 4 months (16 weeks). However, due to reaching the target patient number in a slightly shorter time span, the survey was conducted for a period of 14.5 weeks from 4 May until 12 August 2020.

### Demographics

**Fig. 1:**
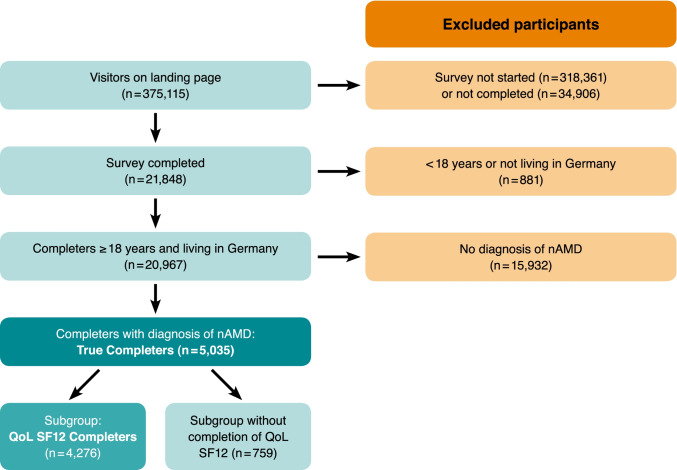
Flowchart of survey participants

Overall, the advertisement for the survey generated 375,115 visits on the landing page (page with first information about the survey, directed after clicking the advertisement). A total of 318,361 visitors did not start the survey and 34,906 surveys had been started but were not completed. In total, 21,848 persons completed the survey out of which 20,967 were older than 18 years and stated to live in Germany. Minors and people not living in Germany were excluded by ending the survey after this information was collected. Out of these, 5035 persons stated that they were having a diagnosis of nAMD; those were defined as “6” and final analyses were based on this population. Out of the True Completers, the optional QoL SF12v2 was fully answered by 4276 nAMD patients, building the subgroup defined as “QoL SF12 Completers” (see Fig. 1).

Analyzing the number of visits on the landing page of the survey via Google Analytics turned out that 60% of the users were accessing the survey via mobile device, 28% via desktop, and 12% via tablet.

Regarding advertisement, the most successful advertisement was Google Display Network, generating 94.1% of all completed surveys. Facebook advertisement generated 3.6% of the completers and Google search engine marketing 2.3%.

### True Completers

**Fig. 2:**
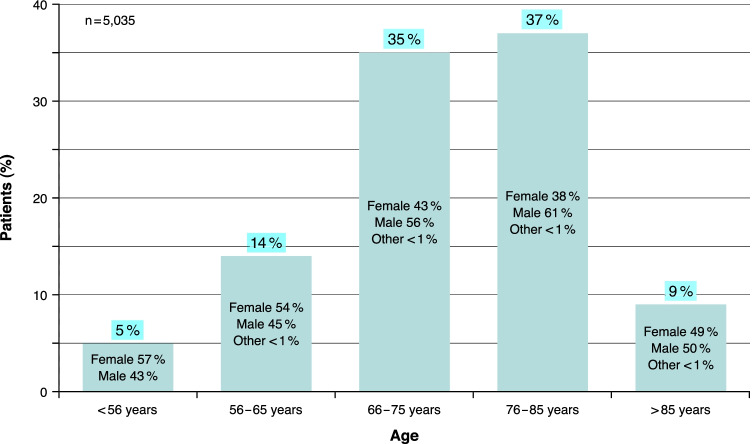
Age distribution of True Completers

A total of 2244 (45%) females and 2781 (55%) males with a diagnosis of nAMD participated in this survey (10 participants stated their gender as “other”). Mean age of all survey participants was 73 ± 10 years, in males 74 ± 9 years and in females 72 ± 11 years. Most participants were in the age groups 76 to 85 years (37%) and 66 to 75 years (35%). There were 14% in the group 56 to 65 years, 5% were younger than 56 years, and 9% were older than 85 years. The distribution pattern among the different age groups almost corresponded to the overall population for both men and women. However, from the age group < 56 years, the proportion of females (57%) decreased with increasing age to 38% females in the group 76–85 years, whereas the group of > 85 years contained a nearly equal proportion of females (49%) and males (50%). For details related to age distribution, see Fig. 2.

A total of 3880 patients (77%) were in a statutory insurance while 1101 patients (23%) had a private insurance (out of those 66.4% had a self-paid private insurance and in 33.6% this insurance was employer-paid). The proportion of males and females with a statutory or a private insurance was similar. Noticeably, the proportion of patients with self-paid private health insurance was particularly high in the age groups 66–75 years (38%) and 76–85 years (40%) compared to the age groups < 56 years (2%) and > 85 years (8%), respectively.

### Disease-related outcomes

The understanding of nAMD from the patient’s point of view is shown in Table 1.

**Table 1:** Understanding of nAMD as assessed by the patient

	Total (*n* = 5035)	Statutory insurance (*n* = 3880)	Private insurance (*n* = 1101)	Treatment with injections (*n* = 2801)	Currently no injections (*n* = 2234)
Very good	11%	9%	14%	13%	8%
Good	32%	31%	35%	37%	26%
Sufficient	33%	33%	33%	33%	32%
Poor	25%	26%	18%	17%	34%

There were no relevant differences regarding the patient’s self-assessed understanding of nAMD related to age and gender. The self-reported understanding of the disease is slightly better in patients having a private health insurance compared to a statutory insurance (49% vs 40% [good or very good understanding]) and better in patients being currently treated with injections compared to currently not receiving injections (50% vs 34%).

In total, 56% of the patients currently received injections into at least one eye. Two-thirds (68%) of all patients were aware of the requirement for regular ophthalmologic examinations and medical treatment, whenever it is necessary. More than half of the patients (60%) knew that the deterioration of their visual performance can be delayed by drug therapy and around half of the study population (54%) knew that there is no cure for nAMD.

**Fig. 3:**
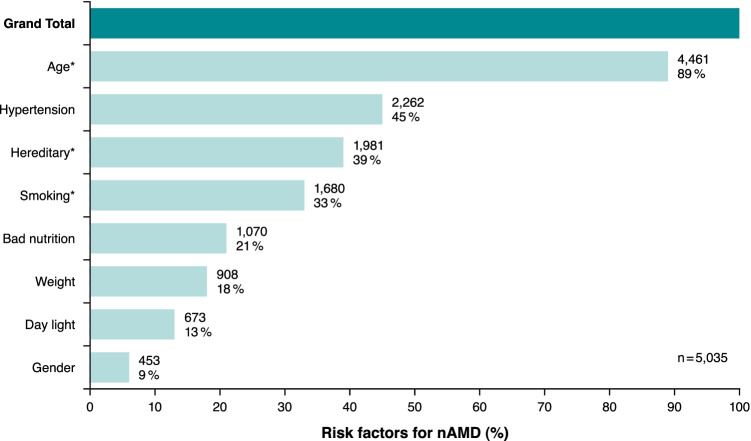
Summary of risk factors for nAMD as stated by the patients (multiple choices allowed); the risk factors confirmed in the literature are marked with an asterisk

The questionnaire asked patients to state risk factors for nAMD from preset response options (multiple choices allowed). Most participants (89%) considered age to be a risk factor for nAMD, followed by hereditary (33%), and smoking (33%). However, some of the risk factors given by the patients were not described and confirmed as strong risk factors in the literature (23, 24), e.g., arterial hypertension (45%), bad nutrition (21%), weight (18%), daylight (13%), and gender (9%). For details, please refer to Fig. 3.

**Fig. 4:**
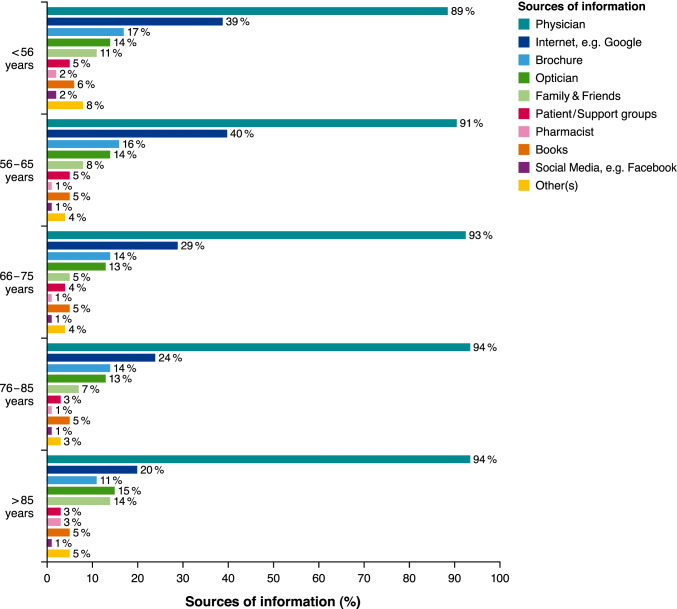
Sources of disease-related information (multiple choices allowed)

Most patients were informed about their disease by their doctor (93%), followed by internet search (29%), flyers (14%), and their opticians (13%). Results on sources of information are displayed in Fig. 4. The distribution according to age groups revealed that younger patients used the internet more frequently than older patients (40% of patients < 56 to 65 years vs 24% of patients in the age group 76–85 years and 20% of patients older than 85 years). There were only slight differences related to gender. More than two-thirds of patients (69%) knew whether or not their diagnosing ophthalmologist was providing the injections. Knowledge about this option increased with age (from 58% in the group < 56 years to 73% in the group > 85 years) and time since diagnosis (from 64% in the group 0–2 months to 71% in the group > 5 years). This could be due to the fact that more older patients received intravitreal injections compared to younger patients (55% in the > 85 years of age group, 61% in the age group 76–85 years, while only 38% of patients < 56 years received injections).

About half of the patients (54%) stated that they had last seen an ophthalmologist within 1 month (mean time since the last visit 6.2 months). While there were no differences concerning the genders in this regard, results suggest a relationship between patients’ age and time since the last visit to an ophthalmologist. In the younger patients’ groups, less patients reported their last ophthalmologist visit within 1 month than in older patients’ groups (in the < 56 years age group 37% and in the 56–65 years age group 44%). A total of 8% of patients noted that their last visit to their ophthalmologist was more than 1 year ago. There were no remarkable differences considering the insurance status (private insurance vs statutory insurance, e.g., last visit less than one month ago was 4% for both; more than 1 year ago was 8% and 9%, respectively).

The majority of patients was able to remember the performed diagnostic methods; however, a relatively large proportion of patients (20%) reported being not sure whether or not optical coherence tomography (OCT) was performed. There were no differences in reported performed diagnostic methods between genders and only small differences concerning the type of health insurance or age groups. However, with increasing age, a slightly increasing proportion of patients reported no fluorescence angiography was performed (33% in the group < 56 years, 44% in the group > 85 years). Diagnostics performed in nAMD as reported by patients are summarized in Table 2.

**Table 2:** Diagnostic methods (*due to rounding total number > 100%)

	All patients (*n* = 5035)		
Diagnostic method	Yes	No	Not sure
Ophthalmoscopy	93%	4%	3%
Visual test using vision boards	91%	4%	5%
Examination of the retina*	89%	5%	7%
Amsler grid test	78%	14%	8%
Optical coherence tomography	69%	11%	20%
Fluorescence angiography	53%	39%	8%

Most of the patients reported a short timespan between occurrence of symptoms and nAMD diagnosis (1–2 months (30%) and 3–6 months (28%)). Although only 3% of patients recorded a time span of 13–21 months, a relatively large number of patients noted that the time span was > 21 months (18%), resulting in a mean time span of 12.8 months ± 20.5 months. Proportions were similar in males and females and between patients with statutory or private insurance. However, when comparing the age groups, it could be seen that younger patients were more likely to be diagnosed early with the disease, compared to older patients. While 41% of patients < 56 years were diagnosed within a time span of 1–2 months between first symptoms and diagnosis, this was true for 27% of patients aged 76–85 years, and for 23% of patients > 85 years, respectively, resulting in a mean time span in the two youngest age groups (< 65 years) of 11 months ± 19.5 months.

The time since diagnosis (until survey participation) ranged from 0–2 months (8%) to more than 6 years (14%). There were only small differences in time since diagnosis when comparing age groups and genders.

Questioned about their symptoms, most patients reported decreased central visual acuity (70%), followed by distorted vision (63%), and increased glare sensitivity (58%, multiple choices allowed). There were no significant differences between genders and between age groups.

A total of 80% of all patients stated that they test their current visual performance on a regular basis at home. Most of these patients were in the age group 66–75 years (83%); patients belonging to the age groups < 56 years or > 85 years reported to carry out the test at home less often (72% and 73%, respectively). More than one-fourth of the study population did not use any aids to check their vision at home (27% in total). Looking at aids to test their visual performance, the majority of patients (81%) stated to use the “Amsler grid”; only a small portion of the patients used vision tests from the internet or via smartphone apps (10% and 8%, respectively). There was a small difference detected; younger patients (age group < 56 years) stated to use vision tests via internet or smart phone app more frequently (18% and 23%, respectively) than older patients (> 85 years with 12% and 12%, respectively).

The mode of transportation to the treating ophthalmologist by age group is displayed in Table 3.

**Table 3:** Transport to the treating ophthalmologist by age group (*due to rounding total number < 100%)

	< 56 years (*n* = 100)	56–65 years (*n* = 353)	66–75 years (*n* = 966)*	76–85 years (*n* = 1142)*	> 85 years (*n* = 240)
Driven by car	55%	57%	58%	52%	53%
Public transport	25%	20%	22%	23%	16%
Driven by taxi	2%	6%	4%	8%	18%
On foot	5%	7%	5%	7%	5%
Self-driving	7%	5%	5%	4%	4%
Picked up	4%	3%	4%	3%	4%
By bicycle	2%	2%	1%	1%	Not specified
Others	Not specified	Not specified	0%	1%	0%

Most patients indicated that they were driven to their ophthalmologist by an accompanying person (55%), followed by public transportation (22%). The proportion of patients driven by taxi is quite small (7%) but slightly increasing with age with a simultaneous decline in public transportation. Approximately the same number of patients reported less than 30 min and less than 1 h travel time to their ophthalmologist (44% and 40%, respectively); however, 4% of all patients needed more than 2 h to get to their doctor. An accompanying person to get to the ophthalmologist was needed by 46% of patients in total. Between genders, a difference could be detected; more female patients (52%) stated that they needed accompaniment than male patients (42%). As it was expected, accompaniment was mostly needed by the eldest patients (> 85 years, 59%) but interestingly also by the youngest patients (< 56 years, 52%).

Most patients were accompanied by close family members such as their wife (40%), husband (28%), and children (24%). Most accompanying persons were female (59%), while only 38% were male. In 23%, the gender was not specified.

Most patients spent 2 h (35%), 1 h (27%), or even less than 1 h (19%) at their ophthalmologist (including waiting time) but there were still patients who were spending 4 or 5 h in their ophthalmologist office (4% and 1%, respectively). There were no differences related to the type of health insurance. Also, regarding age groups, differences in time spent were not concise (26% of younger patients spent less than 1 h at their doctor compared to 17 to 20% in all other age groups).

Fourteen percent of all patients stated that sometimes they needed to cancel an appointment at the ophthalmologist; most often (in 24%) this occurred in younger patients (< 56 years) and in the eldest patients (> 85 years). Reasons for cancellation of an appointment were mostly health-related (60%), followed by vacation (11%). However, a large proportion (32%) of patients did not specify the cause of the cancellation. Health-related cancellations occurred more frequently in females (71%) compared to males (50%) and in younger patients (< 56 years, 71%) compared to the age groups 66–75 years (60%) and 76–85 years (55%). In most of the cases, an alternate appointment within 1 week or 2 weeks was offered to the patients (46% or 36% respectively); only 10% of patients needed to wait more than 3 weeks. There were no relevant differences in relation to gender, age, or type of health insurance.

Patients were questioned about which control and treatment schedule they would prefer: most patients (51%) stated they would prefer treatment as needed (the date for the next injection will be fixed after the results of the control are available, also called treat and extend, T&E) in contrast to 16% of patients that would like a regular appointment every 4 weeks (control every 4 weeks and injection on an “as needed” basis, also called pro re nata, PRN) or a fixed treatment scheme with control and treatment every 4 weeks favored by 15% of patients. More patients in the older age groups (66–75 years with 52%, 76–85 years with 50%, > 85 years with 54%) preferred the treatment as needed compared to the patients of the youngest age group (< 56 years, 43%). Furthermore, the preference of a fixed appointment every 4 weeks decreased slightly with increasing time spent at the ophthalmologist (from 27% in the group < 1 h to 12% in the group of 4 h).

The majority of patients (79%) expressed their wish to receive more information about therapeutic options and the disease in general, less patients about natural disease progression (51%). Around one-fourth of the patients (27%) stated that they feel comfortable with the provided information (multiple entries stating “yes” were possible for this question, but “no” was an exclusive option. Therefore, the presented percentages refer only to “yes” answers). There was no difference regarding genders and age groups. Most patients stated that it is “very important” (46%) or of “utmost importance” (37%) that control visits are close to their home. Only few patients stated that this is “a little important” or “not so important” (10% and 6% respectively). This was expressed by males and females as well as throughout all age groups.

To take part in clinical studies could be an option for 28% of all patients; 41% stated that this is not an option at all, and 31% of patients said they would possibly take part in a clinical study. The willingness to take part in a clinical study seemed to decrease with age (35% of patients < 56 years stated studies as no option vs 54% of patients > 85 years).

Approximately half (54%) of all participants assessed their general health status as “good,” 26% of the patients stated that it was “impaired,” 15% of patients assessed it as “very good” or “excellent,” and only 5% reported a “poor” general health status. This assessment was very similar in both genders but differed within age groups: The proportion of patients stating their general health status as “very good” or “excellent” decreased with increasing age (< 56 years with 23% vs > 85 years with 10%).

However, 31% of the youngest patients (< 56 years) and 47% of these oldest patients (< 85 years) stated that their general health status was “impaired” or “poor,” whereas smaller proportions in the age groups in between reported these general health statuses.

### Quality of life measured by the SF12 questionnaire

To complete the characteristics of patients participating in this survey, a second questionnaire (SF-12) dealing with quality of life (QoL) could be completed voluntarily. The SF12® is a registered trademark of Medical Outcomes Trust and Quality Metric incorporated, and SF12v2® is a Health Survey Standard, Germany [22].

A total of 4276 patients completed this QoL questionnaire. Mean scores and ranges overall were 48.9 (8.0–77.8) for mental health, 43.8 (12.8–68.5) for physical health, and 0.685 (0.35–1.0) for the health utility index. This means that both the mental health and the physical health of the patients are slightly below average results of the test population used to develop those scores (50.0 and 50.1 respectively in the general US population). Calculation of scores is described in detail by Ware et al. [23].

The mean scores and ranges by age are presented in the following table (Table 4).

**Table 4:** Summary and health utility index scores (mean scores and ranges) by age

	< 56 years (*n* = 222)	56–65 years (*n* = 617)	66–75 years (*n* = 1488)	76–85 years (*n* = 1583)	> 85 years (*n* = 366)
Mental component summary	44.2 (12.0–77.8)	47.5 (18.4–68.8)	50.4 (8.1–70.8)	50.1 (12.6–75.5)	47.9 (15.1–71.7)
Physical component summary	47.8 (13.5–66.5)	46.2 (12.8–68.1)	46.3 (15.4–68.5)	43.1 (13.2–63)	36.5 (15.7–59.1)
SF-6D health utility index	0.687 (0.345–1.0)	0.694 (0.357–1.0)	0.717 (0.345–1.0)	0.691 (0.345–1.0)	0.617 (0.345–1.0)

**Fig. 5:**
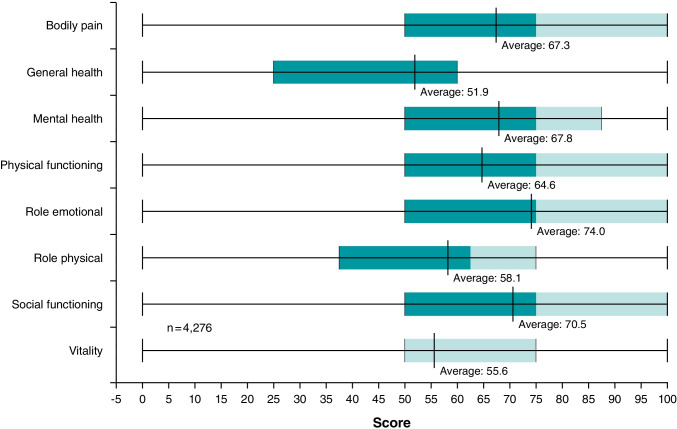
SF-12 raw data scores

In the following figure (Fig. 5), the raw data scores of the eight items (bodily pain, general health, mental health, physical functioning, role emotional, role physical, social functioning, and vitality) are depicted. Patients stated that they feel mostly impaired in general health and role physical.

## Discussion and conclusion

The purpose of this patient survey was to learn more about patients’ awareness of nAMD and how patients get informed about their disease, to identify which factors influence how patients cope with their disease and which kind of information is considered useful from patients’ point of view. An additional objective was to investigate how patients use new technologies, such as the internet, regarding their disease (for example, as a source of information). Data from more than 5000 participants could be analyzed in this survey (True Completers). Among these patients, more males than females took part (55% vs 45%). This is notable, since in AMD survey trials/studies, the gender ratio is often reversed—more women than men filling out surveys on the phone [15] or in private oral interviews [19, 24]. This correlates with the general experience of the authors. The results of the OPTIMA study showing a decreasing proportion of female participants with increasing age might suggest that in older age groups (> 76 years) female patients are more difficult to reach with online surveys than males.

Most of our participants were in the age groups 76 to 85 years (37%) and 66 to 75 years (35%). Overall, three-quarters of patients had a statutory insurance, and one-quarter had a private insurance; particularly in the age groups 66–75 years and 76–85 years, the proportion of patients with self-paid private health insurance was high (38% and 40%, respectively). This is not in line with the general data derived from the association of health insurance companies. Overall, in Germany, 88% of patients have a statutory insurance and about 11% of patients have a private insurance. In our study, we saw a significantly higher number of privately insured patients [25]. These data suggest that patients with private insurance may be better reached by online surveys than patients with statutory insurance. There was no significant difference with regard to waiting, neither time for an appointment nor time spent at the doctor’s office related to the status of insurance indicating equality of treatment.

For most patients, the timespan between occurrence of symptoms and diagnosis of nAMD was 1 to 6 months (58%), which is in accordance with data from the literature [26, 27]. However the delay in diagnosis may also be due in part to patients associating incorrectly nonspecific symptoms with the disease, such as reduced central visual acuity or glare sensitivity, which may not necessarily be caused by nAMD.

Most patients (76%) rated their understanding of the disease as at least sufficient. This finding was confirmed in a study performed by Müller et al. [24], where patients were questioned in 3 subsequent interviews via phone about their views and expectations related to nAMD. More than three-quarters were aware that nAMD may result in vision loss or blindness (83.1%) and a similar proportion (86.4%) was aware that regular monitoring of visual acuity is essential for successful therapy [24].

Looking at the QoL questionnaire, patients stated that they felt mostly impaired in general health and physical activities. This result is in line with data derived from personal performed surveys [28]. The outcome of the QoL questionnaire is reflected in the reasons for cancellation of an appointment with the ophthalmologist as these were mostly health-related (in 60% of patients). It is remarkable that health-related cancellations occurred more frequently in younger patients (< 56 years, 71%) compared to the age groups 66–75 years (60%) and 76–85 years (55%).

Because raising the patient’s level of awareness should increase patient compliance, it is an important observation that more than two-third of the patients (68%) were aware that their disease needs to be controlled on a regular basis and treated on an as needed basis. However, in the study by Müller et al. [24], it turned out that only 16.6% of the patients were aware of the chronic nature of the nAMD disease, and most of the patients hoped or believed that the intravitreal injections were necessary only temporarily. This could be due to the fact that patients participating in online surveys use the internet more regularly and therefore have better access to up-to-date information overall. Since the study of Müller et al. [24] was already conducted in 2017 and internet use has become much more popular since then, there is a possibility that this has also improved the information available to patients.

According to Thomson et al. [15], subjects identified the following strategies to improve therapy adherence: contact with others having the same eye condition, greater education regarding eye disease, and improved transportation services to the clinic.

When asked for potential risk factors for nAMD, most participants were aware of age (89%), but only 39% of hereditary load or 33% of smoking as evidence-based risk factors [29, 30], indicating a need for further information. Especially, smoking was identified as an underestimated risk. This suggests that the perception of this risk is not as widespread among nAMD patients as, for example, among lung cancer patients.

The vast majority (93%) was informed about their disease by their doctor, followed by internet search (29%), brochures (14%), and opticians (13%). A lower percentage of patients were informed by their family and friends (7%), books (5%), and patient support groups (4%). Sources as the pharmacists and social media were only used by 1% of the participants. Younger patients used the internet more frequently than the older ones. Even in the subgroup of patients reporting a very good understanding of disease, the doctor remains the major source of information (95%), with internet (38%), brochures (22%), or patient support groups (7%) with only limited contribution. In this context, internet research seems to serve primarily as an additional source of information, as the physician remains the most important source of information (89–94%) across all age groups and its proportion does not decrease in the same rate as the proportion of internet users in younger age groups increases (20–40%). Because internet users are likely to be rather overrepresented in an online survey, it suggests that the use of the internet as an information source in the real-life population of nAMD patients is probably smaller than our result shows, further highlighting the important role of the ophthalmologist as source of information. Besides other reasons, this might be due to the fact that visual impairment is already more pronounced in patients older than 76 years. Nevertheless, there was a high percentage of old patients using the internet. This trend can be expected to grow continuously, as there is increasing use of the internet, especially in younger patients. There remains a great need for more information related to therapeutic options (79%) and to the nAMD disease in general and regarding progressions (51%) for both genders throughout all age groups.

A total of 56% of the patients currently received intravitreal injections and injections were performed more frequently in patients aged 76 and older. Given that 44% of participants reported not receiving injections, at least currently, it is possible that patients referred the statement “currently” exclusively to the last few days or weeks and therefore reported not receiving injections at present because there was an inactive finding of nAMD at the last examination, and therefore new injections were not directly indicated recently. Furthermore, it is conceivable that patients, although necessary, have not yet received their first injections, or that they are undertreated for other reasons. In addition to reasons listed above, it is also conceivable that not all participants in the OPTIMA survey may actually suffer from nAMD, although all had explicitly stated so. Accordingly, it cannot be excluded with complete certainty that the study population also contains a certain proportion of patients with formerly exudative AMD, untreated exudative AMD, or non-exudative forms.

Neither the insurance status (private or statutory) nor gender or age seems to influence the frequency of visits at the ophthalmologist or the diagnostics, and most patients (81%) test their visual performance on a regular basis at home by using the Amsler grid.

Polat et al. [19] and Droege et al. [14] identified distance to the treatment center as one of the factors, which had the greatest influence on patients’ compliance. This statement was further supported by Boulanger-Scemama [13]: the authors found a statistically significant correlation between poor therapy adherence and a long distance from home to hospital as well as a high age (82.2 years) at start of treatment. These findings from the literature were confirmed by our observations: most patients (regardless of gender and age) pointed out the importance of control visits close to their home. In our survey, almost 90% of patients needed less than 1 h to get to their ophthalmologist; half of the patients were driven by an accompanying person, and about one-quarter used public transportation. Also, in the study by Müller et al. [24] performed by phone interviews, two-thirds of the patients needed a driver or an accompanying person to attend their appointments for intravitreal injections, as car driving is not recommended for patients after treatment. Especially, the comparison with this study indicates a generalizability of the findings of our survey, as our data are comparable to those found out by phone interview.

The difficulty for the patient and/or the accompanying person taking time away from work for the appointments was expressed by Thomson et al. [15]. This is in agreement with the OPTIMA survey results since approximately half of the patients stated that they needed support to get to their ophthalmologist. Cancellations of appointments due to time constraints of the accompanying person were only rarely reported.

Time spent in the ophthalmologist’s office was between 1 to 2 h in most cases. Up to 24% of patients (mostly patients < 56 years and the eldest patients > 85 years) stated that sometimes they need to cancel an appointment at the ophthalmologist due to their health status, other reasons, and vacation. Remarkably, more than one-third of patients did not provide an exact reason for cancellations.

A “treat and extend” regime is the preferred control and treatment schedule in contrast to a fixed appointment every 4 weeks or a treatment on a pro re nata basis. The study by Droege et al. [14] supported the opposite opinion: the authors found that from the patients’ point of view, anxiety of a negative examination result was more pronounced than fear of intraocular injections, which would be an argument for continuous injections rather than for a control and treatment schedule [14]. This is in accordance with results from a discrete choice experiment in which patients accept a high treatment burden to preserve or improve visual function [5].One possible explanation for these partly contradictory results regarding the preferred treatment regimen could be due to patients’ previous positive or negative experiences with one or more of the existing treatment regimens, but these were not captured in our survey.

It is a limitation of this survey due to its internet-based nature that the focus was more on technology-affine and active patients who were already used to the internet. Patients without regular access to a device with internet connection (i.e., phone, tablet, or computer), who tend to gain their knowledge from traditional print media, were most likely not included.

Despite this limitation, the results obtained with our survey appear to be representative of nAMD patients, as the population seem to be well represented in both controlled and observational studies. Therefore, results will help to optimize further patient care and patient-oriented information. In addition, results on the state of patient’s knowledge about their disease and their preferred ways of information acquisition will help to use the internet as a dynamic and sustainable communication channel, both in patient training and in study-related patient support tools.

## Supplementary Information

Below is the link to the electronic supplementary material.

Supplementary file1 (PDF 246 KB)

Supplementary file2 (PDF 252 KB)

## Data Availability

Not applicable.
