# Supplementary material for: nAMD: optimization of patient care and patient-oriented information with the help of an internet-based survey
Source: Graefes Arch Clin Exp Ophthalmol. 2022 May 13;260(10):3241–53. doi: 10.1007/s00417-022-05678-7 (PMC9477947; doi:10.1007/s00417-022-05678-7)
Supplement: Supplementary file 1 — Supplementary file1 (PDF 246 KB) [file 417_2022_5678_MOESM1_ESM.pdf]

## Introductory questions

**1. S1: How old are you?**

| | |
|---|---|
| ......... years | Exclude if < 18 *(go straight to: did not fit)* |

**2. S2: Please choose your sex:**

| | |
|---|---|
| Female | |
| Male | |
| Other | |

**3. S3: Do you currently live in Germany?**

| | |
|---|---|
| Yes | |
| No | Exclude *(go straight to: did not fit* |

**4. S4: Have you ever been diagnosed with wet age-related macular degeneration (AMD) by an ophthalmologist?**

| | |
|---|---|
| Yes | |
| No | Exclude *(go straight to: did not fit)* |

**5. S5: What kind of health insurance do you have?**

| | |
|---|---|
| Statutory health insurance | |
| Private health insurance that I pay for myself | |
| Private health insurance that my employer or my spouse's employer pays in full or in part (e.g. allowance) | |
| Other | |

## Questions about the understanding of the disease

**6. F1: How would you rate your understanding of wet age-related macular degeneration (wet AMD)?**

| | |
|---|---|
| Little | |
| Sufficient | |
| Good | |
| Very good | |

**7. F2: Please select all statements that you think are applicable:**

| | |
|---|---|
| The disease cannot be cured. | |
| The disease must be checked regularly and treated if necessary. | |
| The deterioration in vision can be delayed by drug therapy. | |

8. **F3: Please select all risk factors that you think are applicable for the development of wet age-related macular degeneration (AMD):**

| | |
|---|---|
| Age | |
| Sex | |
| Weight | |
| Smoking | |
| Hereditary burden | |
| Daylight | |
| High Blood pressure | |
| Malnutrition | |

9. **F4: Which symptoms do you have?** *Please select all symptoms that apply.*

**If you experience any of these symptoms, you should schedule an appointment with your eye doctor.**

| | |
|---|---|
| Increased light requirement for vision during the dasy | |
| Enhanced ensitivity to glare(e.g. at night when driving a car) | |
| Increased problems when changing from light to dark and vice versa | |
| Decreased central visual acuity (blurred vision, difficulty reading or recognizing faces) | |
| Distorted vision (straight lines appear bent, e.g. tile joints or picture frames) | |
| Gray or dark spot in the center of the field of vision | |
| None of the above | |

*10.* **F5. Where did you find out more information after the onset of symptoms?**
*Please select all options that apply.*

| | |
|---|---|
| Friends | |
| Family | |
| Pharmacist | |
| Optician | |
| Doctor | |
| Patient oganisations/Self-Help groups | |
| Internet, e.g. Google | |
| Social Media, e.g. Facebook | |
| Brochures | |
| Books | |
| Other | |

*11.* **F6: Which diagnostic measures were carried out on you?** *Please select all options that apply.*

| | Yes | No | Not sure |
|---|---|---|---|
| Eye test using eye charts | | | |
| Amsler grid test | | | |
| Examination of the retina with a special lamp | | | |
| Examination of the fundus after the pupil has been expanded with drops (ophthalmoscopy) | | | |
| Optical coherence tomography (OCT) | | | |
| Fluorescein angiography (a dye is injected into the arm vein, allowing accurate visualization of the retinal vessels) | | | |

**12. F7: Do you regularly check your vision at home?**

| | |
|---|---|
| No | |
| Yes | |

**13. F8: If yes, what tools do you use for this?**

| | |
|---|---|
| Amsler grid | |
| Eye test with smartphone | |
| Eyetest in internet | |
| Other | |
| I don’t use tools | |

**14. F9: Approximately how long was the period between the first symptoms (signs of discomfort) and the diagnosis of wet AMD by the ophthalmologist?**

| | |
|---|---|
| Time period in monmths: ……….. | |

**15. F10: Approximately how many months ago was your last visit to the ophthalmologist??**

| | |
|---|---|
| Number of months:………. | |

**16. F11: Approximately how many months ago were you diagnosed with wet age-related macular degeneration (wet AMD)?**

| | |
|---|---|
| Number of months: ……… | |

**17. F12: Do you know if injections into the eye can be performed at the ophthalmologist who made the diagnosis?**

| | |
|---|---|
| Yes | |
| No | |
| I am not sure | |

**18. F13: Are you currently receiving injections (shots) in the eye due to wet age-related macular degeneration (wet AMD):**

| | |
|---|---|
| No | |
| Yes | |

**19. F14: How do you get to the injecting ophthalmologist mostly?**

| | | |
|---|---|---|
| Public transportation | | |
| By Taxi | | |
| By Foot | | |
| By Bike | | |
| By car: I drive myself | | |
| By car: I’m driven | | |
| I am picked up by the practice | | |
| Other | | |

**20. F15: Do you need a person to accompany you to the injecting ophthalmologist?**

| | |
|---|---|
| No | |
| Yes | |

**21. F16 Who is accompanying you?** *Please select all persons, who sometimes accompany you.*

| | |
|---|---|
| Husband | |
| Wife | |
| Daughter | |
| Son | |
| Daughter in law | |
| Son in law | |
| Acquaintance | |
| Care service | |
| Other person | |

**22. F17: How much time do you need to get from your home to the injecting ophthalmologist?**

| | |
|---|---|
| Under 30 minutes | |
| Under one hour | |
| Under 2 hours | |
| Over 2 hours | |

**23. F18: How much time do you spend at the injecting ophthalmologist (including waiting time)?**

Time in hours:

| | |
|---|---|
| Less than one hour | |
| 1 | |
| 2 | |
| 3 | |
| 4 | |
| 5 | |
| 6 | |
| 7 | |

**24. F19: Have you occasionally had to cancel scheduled appointments with the injecting ophthalmologist?**

| | |
|---|---|
| No | |
| Yes | |

**25. F20: What were the reasons for your appointment cancellations with the injecting ophthalmologist?** *Please select all reasons that apply.*

| | |
|---|---|
| My accompanying person did not have time | |
| Problems with the journey | |
| Health related | |
| Fear of injection (syringe) | |
| Vacation | |
| Other reasons | |

**26. F21: How quickly do you usually get a rescheduled appointment with the injecting ophthalmologist?**

| | |
|---|---|
| Within 1 week | |
| Within 2 weeks | |
| Within 3 weeks | |
| After more than 3 weeks | |

## Questions about patient needs

**27. F22: Which control and treatment regimen would you prefer if you had the choice of?**

| | |
|---|---|
| Fixed appointment every 4 weeks for control and injection | |
| Fixed appointment every 8 weeks for control and injection | |
| Fixed appointment every 12 weeks for control and injection | |
| Fixed appointment every 4 weeks for control; injection only when necessary (is called PRN) | |
| An appointment for the next injection is set according to the result of the control visit (is called Treat and Extend) | |

***28.*** **F23: Would you like more information about your disease?** *Please select all areas, where you would like more information.*

| | |
|---|---|
| Yes, about the disease and ist course | |
| Yes, about therapy options | |
| Yes, about examination methods | |
| Yes, about prevention | |
| Yes, about self-control options | |
| Yes, about aids and auxiliary services | |
| Yes, about patient organisations | |
| No, I am sufficiently informed | (Exclusive Option) |

**29. F24: How important is it for you that your check-ups take place close to home?**

| | |
|---|---|
| Absolutely necessary | |
| Very important | |
| A little important | |
| Not so important | |

**30. F25: Could you imagine participating in a clinical trial testing a drug for age-related macular degeneration (AMD)?**

*Information: A clinical trial is a research study in which participants receive a treatment that is often not yet publicly available, such as a drug that is being developed. The investigating physicians and researchers are trying to determine the safety and efficacy of the treatment by collecting data while participants are using it. For example, researchers might give a drug to participants with AMD to see if that drug helps with symptoms. To do this, participants in a research study must visit the study center regularly for medical exams.*

| | |
|---|---|
| No, in no case | |
| Rather no | |
| Maybe | |
| Rather yes | |
| Yes definitely | |

**31. SF: We would like to ask you further questions about your assessment of your state of health. This questionnaire contains a further 12 questions. Are you ready to answer these questions?**
*Answering these questions is voluntary.*

| | |
|---|---|
| Yes | |
| No | |

If yes => SF-12 questionnaire
