# Supplementary material for: nAMD: optimization of patient care and patient-oriented information with the help of an internet-based survey
Source: Graefes Arch Clin Exp Ophthalmol. 2022 May 13;260(10):3241–53. doi: 10.1007/s00417-022-05678-7 (PMC9477947; doi:10.1007/s00417-022-05678-7)
Supplement: Supplementary file 2 — Supplementary file2 (PDF 252 KB) [file 417_2022_5678_MOESM2_ESM.pdf]

Einleitungsfragen

1. S1: Wie alt sind Sie?

……… Jahre	**Exclude if < 18** *(go straight to: did not fit)*

2. S2: Bitte wählen Sie Ihr Geschlecht aus:

Weiblich	
Männlich	
Anderes	

3. S3: Leben Sie derzeit in Deutschland?

Ja	
Nein	**Exclude** *(go straight to: did not fit*

4. S4: Wurde bei Ihnen jemals eine feuchte altersbedingte Makuladegeneration (AMD) durch einen Augenarzt diagnostiziert?

Ja	
Nein	**Exclude** *(go straight to: did not fit)*

5. S5: Welche Art von Krankenversicherung haben Sie?

Gesetzliche Krankenversicherung	
Private Krankenversicherung, die ich selbst bezahle	
Private Krankenversicherung, die mein Arbeitgeber oder der Arbeitgeber meines Ehepartners vollständig oder teilweise bezahlt (zum Beispiel Beihilfe)	
Sonstiges	

Fragen zum Krankheitsverständnis

6. F1: Wie würden Sie Ihr Verständnis der <u>feuchten altersbedingten Makuladegeneration</u> (feuchte AMD) einschätzen?

Wenig	
Ausreichend	
Gut	
Sehr gut	

7. F2: Bitte wählen Sie alle Ihrer Meinung nach zutreffenden Aussagen aus:

Die Erkrankung ist nicht heilbar.	
Die Erkrankung muss regelmäßig kontrolliert und bei Bedarf behandelt werden.	
Die Sehverschlechterung kann durch eine medikamentöse Therapie verzögert werden.	

8. F3: Bitte wählen Sie alle Ihrer Meinung nach zutreffenden Risikofaktoren für die Entstehung der feuchten altersabhängigen Makuladegeneration (AMD) aus:

Alter	
Geschlecht	
Gewicht	
Rauchen	
Erbliche Belastung	
Tageslicht	
Bluthochdruck	
Fehlernährung	

9. F4: Welche Symptome haben Sie? *Bitte wählen Sie alle zutreffenden Symptome aus.*

Wenn sie eines dieser Symptome bei sich feststellen, sollten sie einen Augenarzttermin vereinbaren.

Tagsüber erhöhter Lichtbedarf beim Sehen	
Verstärkte Blendempfindlichkeit (z.B. nachts beim Autofahren)	
Verstärkte Probleme beim Wechsel von Hell nach Dunkel und umgekehrt	
Verminderte zentrale Sehschärfe (verschwommenes Sehen, Schwierigkeiten beim Lesen oder Erkennen von Gesichtern)	
Verzerrtes Sehen (gerade Linien erscheinen verbogen, z.B. Kachelfugen oder Bilderrahmen)	
Grauer bzw. dunkler Fleck in der Mitte des Gesichtsfeldes	
Keines der genannten Symptome (exclusive option)	

***10.* F5. Wo haben Sie sich nach Auftreten der Symptome informiert?**

Bitte wählen Sie alle zutreffenden Optionen aus.

Freunde	
Familie	
Apotheker	
Optiker	
Arzt	
Patientenorganisationen/Selbsthilfegruppen	
Internet, z. B. Google	
Social Media, z.B. Facebook	
Broschüren	
Bücher	
Sonstige	

***11.* F6: Welche diagnostischen Maßnahmen wurden bei Ihnen durchgeführt?** *Bitte wählen Sie alle zutreffenden Optionen aus.*

	Ja	Nein	Unsicher
Sehtest anhand von Sehtafeln			
Amsler-Gitter-Test			
Untersuchung der Netzhaut mit einer speziellen Lampe			
Untersuchung des Augenhintergrundes nachdem die Pupille mit Tropfen weit gestellt wurde (Ophthalmoskopie)			
Optische Kohärenztomographie (OCT)			
Fluoreszensangiographie (in die Armvene wird ein Farbstoff eingespritzt, dadurch wird eine genaue Darstellung der Netzhautgefäße möglich)			

12. F7: Überprüfen Sie zu Hause regelmäßig Ihre Sehkraft?

Nein	
Ja	

13. F8: Wenn ja, welche Hilfsmittel nutzen Sie dafür?

Amsler-Gitter	
Sehtest mit Smartphone	
Sehtest im Internet	
Andere	
Ich benutze kein Hilfsmittel	(Exclusive)

14. F9: Wie lange war ungefähr der Zeitraum zwischen ersten Symptomen (Anzeichen von Beschwerden) und der Diagnosestellung feuchte AMD durch den Augenarzt?

Zeitraum in Monaten: ………..	

15. F10: Vor wie vielen Monaten waren Sie ungefähr zuletzt beim Augenarzt?

Anzahl der Monate:……….	

16. F11: Vor wie vielen Monaten wurde bei Ihnen ungefähr eine <u>feuchte</u> altersbedingte Makuladegeneration (feuchte AMD) diagnostiziert?

Anzahl der Monate: ………	

17. F12: Wissen Sie, ob bei dem Augenarzt, der die Diagnose gestellt hat, auch Spritzen in das Auge vorgenommen werden können?

Ja	
Nein	
Ich bin nicht sicher	

18. F13: Bekommen Sie momentan Injektionen (Spritzen) ins Auge aufgrund einer <u>feuchten altersbedingten Makuladegeneration (feuchten AMD):</u>

Nein	
Ja	

19. F14: Wie kommen Sie überwiegend zum injizierenden Augenarzt?

Öffentlicher Nahverkehr		
Mit dem Taxi		
Zu Fuß		
Mit dem Fahrrad		
Mit dem Auto: Ich fahre selbst		
Mit dem Auto: Ich werde gefahren		
Ich werde von der Praxis abgeholt		
Sonstiges		

20. F15: Benötigen Sie eine Begleitperson zum injizierenden Augenarzt?

Nein	
Ja	

21. F16 Wer ist die Begleitperson? *Bitte wählen Sie alle Personen aus, die Sie manchmal begleiten.*

Ehemann	
Ehefrau	
Tochter	
Sohn	
Schwiegertochter	
Schwiegersohn	
Bekannte/er	
Pflegedienst	
Andere Person	

22. F17: Wieviel Zeit benötigen Sie, um von zu Hause zum injizierenden Augenarzt zu kommen?

Unter 30 Minuten	
Unter einer Stunde	
Unter 2 Stunden	
Über 2 Stunden	

23. F18: Wieviel Zeit verbringen Sie beim injizierenden Augenarzt (einschließlich Wartezeit)?

Zeit in Stunden:

Weniger als 1 Stunde	
1	
2	
3	
4	
5	
6	
7	

24. F19: Mussten Sie gelegentlich vereinbarte Termine beim injizierenden Augenarzt absagen?

Nein	
Ja	

25. F20: Was waren die Gründe für Ihre Terminabsagen beim injizierenden Augenarzt? *Bitte wählen Sie alle zutreffenden Gründe aus.*

Meine Begleitperson hatte keine Zeit	
Probleme mit der Anreise	
Gesundheitsbedingt	
Angst vor der Injektion (Spritze)	
Urlaub	
Andere Gründe	

26. F21: Wie schnell erhalten Sie in der Regel einen Ersatztermin beim injizierenden Augenarzt?

Innerhalb von einer Woche	
Innerhalb von 2 Wochen	
Innerhalb von 3 Wochen	
Nach mehr als 3 Wochen	

Fragen zu Patientenbedürfnissen

27. F22: Welches Kontroll- und Behandlungsschema würden Sie bevorzugen, wenn Sie die Wahl hätten?

Fester Termin alle 4 Wochen zur Kontrolle und Spritzengabe	
Fester Termin alle 8 Wochen zur Kontrolle und Spritzengabe	
Fester Termin alle 12 Wochen zur Kontrolle und Spritzengabe	
Fester Termin alle 4 Wochen zur Kontrolle; Spritzengabe aber nur wenn nötig (wird als PRN bezeichnet)	
Ein Termin zur nächsten Spritzengabe wird nach dem Ergebnis der Kontrolle festgelegt (wird als Treat und Extend bezeichnet)	

28. **F23: Würden Sie sich mehr Informationen über Ihre Erkrankung wünschen?** *Bitte wählen Sie alle Bereiche aus, zu denen Sie sich mehr Informationen wünschen.*

Ja, zur Erkrankung und deren Verlauf	
Ja, zu Therapiemöglichkeiten	
Ja, zu Untersuchungsmethoden	
Ja, zur Vorbeugung	
Ja, zu Selbstkontrollmöglichkeiten	
Ja, zu Hilfsmitteln und Hilfsleistungen	
Ja, zu Patientenorganisationen	
Nein, ich bin ausreichend informiert	(Exclusive Option)

29. F24: Wie wichtig ist es für Sie, dass Ihre Kontrolluntersuchungen in der Nähe Ihres Wohnortes stattfinden?

Absolut notwendig	
Sehr wichtig	
Ein bisschen wichtig	
Nicht so wichtig	

30. F25: Könnten Sie sich vorstellen an einer klinischen Studie teilzunehmen, in der ein Medikament bei altersbedingter Makuladegeneration (AMD) getestet wird?

Information: Eine klinische Studie ist eine Forschungsstudie, bei der die Teilnehmer eine Behandlung erhalten, die oft noch nicht öffentlich erhältlich ist, z. B. ein Medikament, das gerade entwickelt wird. Die untersuchenden Ärzte und Forscher versuchen, die Sicherheit und Wirksamkeit der Behandlung durch die Erhebung von Daten während der Anwendung bei den Teilnehmern zu bestimmen. Zum Beispiel könnten die Forscher Teilnehmern mit AMD ein Medikament verabreichen, um zu sehen, ob dieses Medikament bei den Symptomen hilft. Die Teilnehmer einer Forschungsstudie müssen das Studienzentrum dazu regelmäßig zu medizinischen Untersuchungen besuchen.

Nein, auf keinen Fall	
Eher nein	
Vielleicht	
Eher ja	
Ja, unbedingt	

31. SF: Gerne möchten wir Ihnen weitere Fragen zu Ihrer Beurteilung Ihres Gesundheitszustandes stellen. Dieser Fragebogen beinhaltet weitere 12 Fragen. Sind Sie bereit diese Fragen zu beantworten?

Die Beantwortung dieser Fragen ist selbstverständlich freiwillig.

Ja	
Nein	

Wenn ja => SF-12 Fragebogen.
